# Word learning and lexicalization in a second language: Evidence from the Prime lexicality effect in masked form priming

**DOI:** 10.3758/s13421-022-01274-6

**Published:** 2022-02-10

**Authors:** Shusaku Kida, Joe Barcroft, Mitchell Sommers

**Affiliations:** 1 Doshisha University, Kyoto, Japan; 2 Washington University in St. Louis, St. Louis, MO USA

**Keywords:** Prime lexicality effect, Masked form priming, New word lexicalization, Second language vocabulary learning, Semantic and form processing

## Abstract

In a masked form priming lexical decision task, orthographically related *word* primes cause null or inhibitory priming relative to unrelated controls because of lexical competition between primes and targets, whereas orthographically related *nonword* primes lead to facilitation because nonwords are not lexically represented and hence do not evoke lexical competition. This *prime lexicality effect* (PLE) has been used as an index of new word lexicalization in the developing lexicon by using to-be-learned words and their orthographic neighbors as primes and targets, respectively. Experiment 1 confirmed an inhibitory effect of −46 ms among native English speakers and faciliatory effects of 52 ms by Japanese English learners without critical word training. In Experiment 2, Japanese English learners studied novel English words while performing a meaning-based, form-based, or no task during learning. Recall measures indicated a dissociation between these two types of processing, with a form-based task leading to greater recall of L2 words and a meaning-based task leading to greater recall of L1 words. Results indicated that all three learning conditions produced neither facilitation nor inhibition (null priming effect). Taken together, the results of the two experiments demonstrate that the PLE can occur in a second language (L2) and that the training procedure can yield at least partial lexicalization of new L2 words.

Recent research on new word learning distinguishes two stages of vocabulary acquisition—lexical configuration and lexical engagement (Leach & Samuel, 2007). *Lexical configuration* refers to an initial developmental stage in which learners store factual information about a word, such as the form or meaning in episodic memory, whereas *lexical engagement* refers to a relatively later stage of development in which the lexical information is established and represented in the mental lexicon and the newly established representation dynamically interacts with other already existing representations (lexicalization). These two stages differ in terms of one’s lexical development with lexical engagement being perhaps the more “sought after” index of vocabulary acquisition (Qiao & Forster, 2013).

One methodology that researchers have used to assess degree of lexical engagement for newly learned word forms is the masked form priming lexical decision paradigm (e.g., Qiao & Forster, 2013, 2017; Qiao et al., 2009). In this paradigm, participants are asked to make lexical decisions to target items that are preceded by prime words presented very briefly so that participants are typically unaware of the prime words’ identity. On the one hand, when *nonword* primes are used, facilitatory priming is typically observed (Forster & Davis, 1984; Forster, et al., 1987; Forster et al., 2003; Forster & Veres, 1998), especially when primes and targets are short and low-neighborhood (*N*) words (Davis & Lupker, 2006) or when they are very long words (Qiao & Forster, 2013). On the other hand, when *word* primes are used, due to the lexical competition between the prime and target, the facilitatory priming diminishes to null or even sometimes causes inhibitory priming when primes are higher in frequency than targets (Forster & Veres, 1998) or when primes and targets are high-*N* words (Nakayama et al., 2008). This discrepancy between the direction of priming for nonword (facilitative priming) and word (null or inhibitory priming) primes is known as the *prime lexicality effect* (PLE; please also see Davis & Lupker, 2006, for additional considerations regarding constraints and parameters related to obtaining a PLE).

To date, the PLE has been utilized to investigate adult first language (L1) word learning (Bowers et al., 2005; Qiao & Forster, 2013; Qiao et al., 2009). Qiao et al. (2009), for example, had L1 English participants learn pseudowords (e.g., *banara*) that were similar in form to real words. They then conducted a masked form priming LDT experiment in which targets included real words known to be in the L1 lexicon (e.g., *banana*) and that were similar in form to the pseudoword primes. Half the primes were the trained pseudowords and half were novel pseudowords. The results indicated that an equal level of facilitative priming effect was observed for both the trained and untrained primes, suggesting that these pseudowords were not lexicalized in the lexicon. Qiao and Forster (2013) asked participants to learn 48 pseudowords (e.g., *baltery*) by presenting both definitions and pictures. The to-be-learned pseudowords were neighbors of real words (e.g., *battery*). Similar to the study by Qiao et al. (2009), half of the pseudowords used as primes were trained, whereas the other half were not. The results of the masked form priming LDT revealed that both trained and untrained primes showed facilitative priming. They then trained another group of participants on the same set of words four times over a period of 2 weeks and compared their performance with the untrained participants. In this second experiment, a clear difference was found between trained and untrained participants. There was no significant difference between related and unrelated prime conditions for trained participants (i.e., null priming effect), whereas there was a significant faciliatory effect for related primes in the untrained participants. Qiao and Forster suggested that the different findings for trained versus untrained participants represented a PLE.

In L2 word learning, Elgort (2011) asked participants to learn 48 pseudo L2 words in Experiment 1. In the learning session, the to-be-learned words were presented both in the spoken and written mode with their meanings, grammatical information, and example sentences. They were instructed to learn each word and also to repeat it aloud. Furthermore, they were asked to learn these words through a set of word cards in their home both in the form-to-meaning and meaning-to-form directions for 1 week. Subsequently, the masked form priming LDT was administered. Note that in this study, the duration of prime presentation was 522 milliseconds (ms), allowing the prime to be visible. The LDT had three prime conditions: related *trained* pseudoword primes (e.g., *teometry–GEOMETRY*), related *untrained* nonword primes (e.g., *geobetry–GEOMETRY*), and unrelated (control) untrained word primes (e.g., *abdicate–GEOMETRY*). The results indicated that the related untrained nonword primes caused significant facilitative priming, whereas the related trained pseudoword primes did not show significant priming, though numerically 20 ms facilitative effect was observed. Elgort suggested that the absence of faciliatory priming for the trained primes indicated that the participants had learned these pseudowords and that they had become lexicalized in the participants’ mental lexicons.

Replicating their previous experiment with L1 native speakers of English (Qiao & Forster, 2013) in which facilitatory priming disappeared over the four learning sessions, Qiao and Forster (2017) examined new L2 word lexicalization with the typical masked form priming procedure. The results demonstrated that, in contrast to their previous experiment with L1 English speaker, L2 English learners produced facilitatory priming over time. Qiao and Forster (2017) argued that L2 lexicon is stored in a different way compared with L1 lexicon. These results are consistent with those of Nakayama and Lupker (2018), who examined whether Japanese English learners’ lexical competition takes place between previously known primes and targets in L2, and found that, in a typical masked form priming procedure, facilitatory priming was repeatedly observed. The only hint of the lexical competition they found was in error rates rather than reaction times in the unmasked LDT, suggesting that different-script L2 learners of English deal with L2 orthographic similarity differently than do L1 native English speakers.

## The present study

The goal of the present study was to further examine lexicalization of new L2 words using the PLE paradigm. Specifically, the study investigated how different types of processing and learning affect lexicalization of new L2 words given that previous research has yet to examine this issue. Studies on L2 vocabulary acquisition have revealed that different types of vocabulary-related processing result in different types of learning in a dissociable manner (e.g., Barcroft, 2002, 2003, 2009; Kida, 2020; Sommers & Barcroft, 2013; Wong & Pyun, 2012). Such findings are consistent with the predictions of the type of processing resource allocation (TOPRA) model for L2 vocabulary learning (Barcroft, 2002) that posits that we allocate limited processing resources to L2 word form or meaning according to the demands imposed by the learning task. As a result, increased processing for one aspect of word learning results in increased memory for that aspect while simultaneously decreasing processing and learning of other aspects of word learning, at least when overall processing demands are sufficiently high. For example, when a learner allocates processing resources to the semantic component of a word by performing a semantic task whose processing demands are sufficiently high, the semantic information (e.g., L1 translation in a L2-and-L1 paired association learning) is better encoded and retained. However, this same increase in semantic processing can deplete remaining resources that otherwise could be used to process the structural (formal) aspects of novel words, such as their spelling and therefore, the learner does not learn the spelling of the L2 word as well. Conversely, when learners allocate more resources to structural or form-based components by performing form-based learning tasks, L2 word form or other structural components is enhanced, but at the expense of encoding and retention of semantic components if, again, overall processing demands are sufficiently high.

In the case of new L2 word learning, word forms are not known to the learners, but their basic meanings tend to be understood through their L1-based semantic information, such as with L1 translations (Coady et al., 1993; Jiang, 2000). In other words, during the initial stages of L2 vocabulary learning, semantic information tends to be known by learners (without denying the need to tune L2-specific semantic space over time) while they learn the novel L2 word forms that correspond to known semantic information gradually over time. Thus, a speaker of L1 English tasked with learning the L2 Spanish word *durazno* (*peach*) has considerable semantic information through their L1 and therefore must map the new word form (durazno) onto an already existing semantic representation (peach). In this light, what is oftentimes important when learning new L2 words in the initial stages is not to (re)learn word *meaning* but to encode and retain the novel *form* of words since (a) L2 word forms and (b) their initial-stage (unrefined) L1-based meanings are dissociable components of initial-stage L2 vocabulary learning (Barcroft, 2002). In the current study, we compared lexicalization of L2 vocabulary learning following either semantically based or formally based learning tasks to determine whether differential attention to meaning versus form might lead to different levels of new word lexicalization.

In the following sections, we report two separate experiments. The first compares masked form priming LDT data from native English L1 participants and L1 Japanese individuals using highly familiar (for the English L1 participants) English words as targets. Critically, none of the English prime words were known to the L1 Japanese participants. We predicted that we would observe null or inhibitory priming from the L1 English participants, as the primes and targets should compete given that these were all well-known words to the L1 English participants, and also they were borrowed from previous studies which showed inhibitory priming by L1 English speakers (Davis & Lupker, 2006; Nakayama et al., 2008). In contrast, we expected facilitative priming from the L1 Japanese speakers as the prime words were unfamiliar to them and there was no training prior to the LDT task.

Experiment 2 was another masked form priming experiment with the same stimuli and procedure as the first experiment. In this case, however, participants were trained on the to-be-learned L2 words (i.e., prime words) either in a semantic processing condition, a form processing condition, or a control condition. We included four types of recall tests (L2 free recall, L1 free recall, L1-to-L2 cued recall, and L2-to-L1 cued recall) in order to examine (a) the effects of semantic and form processing on the L2 word learning at the lexical configuration level and (b) whether the semantic and form processing tasks yielded what would be expected from the perspective of the TOPRA model. The two free recall measures were particularly important as a means to measure memory for new L2 word forms and meanings. L2 free recall measured their memory for new L2 word forms while L1 free recall measured learners’ memory for the to-be-learned word meanings (L1 translations). In consideration of TOPRA predictions, we expected performance for the semantic processing condition to be better when compared with that of the form processing condition for L1 free recall and the converse for L2 free recall. Furthermore, Experiment 2 included masked form priming LDT to examine the effects of semantic and form processing on the L2 word learning at the lexical engagement level. To the extent that training led to lexicalization of new L2 words, we expected reduced priming effects compared with those obtained from untrained Japanese learners of English from Experiment 1, that is, for the PLE to emerge for these L2 learners. In consideration of predictions of the TOPRA model, we noted that the PLE might be observed more clearly in the form learning condition than in the semantic learning condition because the former condition should lead to more novel word form encoding and form-meaning mapping overall. Alternatively, however, if emergence of the PLE as a measure or indication of lexicalization is somehow tied to the relative strength or robustness of the semantic aspects of a newly developing lexical entry, it is possible that the semantic group might exhibit the effect sooner when compared with the form group, the control group, or both.

In summary, the present investigation advances prior research on lexicalization of L2 words, by examining whether learning types, independent of overall amount of vocabulary learning, would impact the extent to which lexicalization occurs. In order to examine the possibility of a different pattern between amount of vocabulary learning and amount of lexicalization, the study included two different learning tasks, one focused on meaning and the other on form, that have been found in previous research (Barcroft, 2002) to affect amount of vocabulary learning differently. Would those two different learning tasks lead to a similar pattern when it comes to amount of lexicalization? The present study was designed to answer this question by first confirming the previous findings of Barcroft demonstrating more word form recall for structural over semantic elaboration and then by assessing whether or not patterns in lexicalization would follow this same pattern.

## Experiment 1

### Method

#### Participants

Participants in Experiment 1 were 62 adult native speakers of English and 48 Japanese University learners of English. Native speakers of English were recruited from the participant pool in a university in the USA. Data provided by eight Japanese students were excluded because their error rates on the LDT were over 20%. Data from three additional Japanese participants were excluded because they did not complete the experiment. Data from the remaining 37 Japanese participants are included in the current analyses. The questionnaire on Japanese English learners’ backgrounds revealed that most of them started learning English when they were 12 or 13 years old and that they had at least 6 years of formal instruction in English. This is a typical background of English learning for Japanese university students. Their scores on the Test of English for International Communication (TOEIC), developed by Educational Testing Service, suggest that their English proficiency was intermediate. The background information of the Japanese participants appears in Table 1.

**Table 1:** Background information of Japanese participants in Experiment 1 (*n* = 37)

	Mean	*SD*	Minimum	Maximum
Age	18.65	0.63	18	20
Age began learning English	12.41	1.19	9	13
Years of formal instruction	6.43	0.87	6	10
TOEIC score	415.78	85.15	240	590
Self-rating:				
Speaking	2.73	1.02	1	5
Listening	3.24	1.28	1	6
Reading	3.81	1.56	1	7
Writing	3.41	1.42	1	7

Self-rating score is on a scale from 0 (*minimum proficiency*) to 10 (*near-native proficiency*). TOEIC (Test of English for International Communication) is a standardized English proficiency test developed by Educational Testing Service, whose score ranges from 10 to 990.

#### Power analysis

A power analysis was performed in R using the *lavaan* package (Rosseel, 2012). We generated 1,000 datasets with sample sizes ranging between 15 and 150 to determine power to detect effects in each condition. Simulations revealed that a sample size of 27 in each condition allowed for the detection of small to moderate (.2 or larger) effects with 80% power.

#### Stimuli and design of the experiment

Word pairs (24 target words and their corresponding 24 form-related prime words) in the related condition of the masked form priming LDT were taken from previous studies (Davis & Lupker, 2006; Nakayama et al., 2008) that demonstrated the inhibitory masked priming effect in L1 visual word recognition (e.g., *omen*–*OPEN*). Because prime words were used as the to-be-learned words in the learning session in Experiment 2, it was necessary to select word pairs whose prime words were unfamiliar to L2 learners. Therefore, word pairs whose prime words (mono-morpheme words) were of low frequency (under 100 in SUBTLEX in the English Lexicon Project database; Balota et al., 2007) and not included in an English word familiarity database of 3,000 high-frequency English words for Japanese English-as-a-foreign-language (EFL) learners (Yokokawa, 2006) were selected. It was also confirmed that the position of letter substitution between the target words and prime words was balanced such that substitution occurred at the beginning position for eight words, middle position for another eight words, and end position for the other eight words.^1^

For 24 unrelated control word primes (e.g., *flux*–*OPEN*), words whose number of letters, *N*-size, and frequency were similar to the corresponding related prime words were selected, using the English Lexicon Project database (Balota et al., 2007). Letters in the control primes differed from targets at all positions (i.e., there was no overlap in the component letters of primes and targets in the control condition).

For nonword targets and their primes in the LDT, nonwords whose *N*-size was similar to corresponding word targets were selected. Additionally, form-related nonword primes were created by substituting one letter for another letter. The position of the substitution was the same as in the corresponding word pairs. It was confirmed that the *N*-size of the created nonword primes was the same as that of the corresponding word primes. Based on this procedure, 24 target nonwords and their nonword primes (related and control primes) were selected.

Two separate lists were created for the purpose of counterbalancing. Lexical characteristics between the two lists were matched as closely as possible in terms of target word characteristics and related prime word characteristics (see Table 2). The participants were randomly assigned into one of the two list conditions. Participants judged each word only once in the LDT, but across lists, all words were presented in the two conditions.

**Table 2:** Lexical characteristics for target and related prime words in the lexical decision task

	Target words				Prime words		
	NL	*N*	Frequency	Familiarity	NL	*N*	Frequency
List 1	4.50 (0.50)	6.33 (5.02)	163.64 (192.96)	5.81 (0.68)	4.50 (0.50)	5.50 (3.43)	1.96 (1.87)
List 2	4.50 (0.50)	6.58 (4.61)	175.73 (213.29)	5.85 (0.82)	4.50 (0.50)	5.83 (4.86)	1.57 (1.66)

NL refers to the number of letters, *N* refers to neighborhood size, Frequency is based on SUBTLEX by the English Lexicon Project database, Familiarity refers to English word familiarity ratings by Japanese learners of English based on Yokokawa (2006). Familiarity for prime words were not available because they were too low for frequency level. Standard deviations are shown in parentheses.

#### Apparatus and procedure

The DMDX software (Forster & Forster, 2003) was used for the presentation of stimuli and measurement of RTs and error rates. In the masked form priming LDT, the participants were instructed to judge whether the presented target letter strings were an English word or not and to do so as rapidly and accurately as possible. Before the main session, the participants took part in a practice session. It consisted of eight trials in which four words and four nonwords were shown. In the LDT, a row of hash marks (#####), the prime and the target word were presented sequentially. The hash marks were used as a forward mask, and the target word functioned as a backward mask. The presentation duration of the forward mask and the prime were 800 ms and 67 ms,^2^ respectively. The prime was presented in lowercase and the target word was shown in uppercase for 2,500 ms or until the participants made a response. All stimuli were presented on a black screen in white Courier 36-pt font. After the LDT, Japanese participants were presented 48 English words and their Japanese translations, of which 24 words were the primes in the LDT. They were asked to indicate words they knew or thought they knew. Any word indicated was not included in the analyses.

### Results

In both experiments, analyses were conducted on response latencies only for the correct responses for word trials. As is typical for lexical decision experiments, error rates were extremely low; therefore we did not include analyses of the error data. RTs longer than 1,500 ms were treated as outliers and excluded (46 out of 799 observations which were  was 1.94%). As described above, data provided by Japanese participants whose error rates were more than 20% in the LDT and who did not complete all of the experimental procedures were excluded from the analyses. Some data from Japanese participants were excluded from the analyses when they indicated that they had prior knowledge about the prime word (16 observations among 2,136 possible responses, 0.75%). The descriptive statistics for the results of the masked form priming LDT with the raw RT data appear in Table 3. To determine if there were any speed–accuracy trade-offs, we conducted one-way analyses of variance (ANOVAs) on the accuracy measures in both groups for the word and nonword targets. All *p* values were greater than .15 (lowest *p* value was .16 for the word target condition for L1 English speakers), suggesting that differences in latencies were not significantly affected by differences in accuracy.

**Table 3:** Descriptive statistics for the results of native English speakers (*n* = 62) and Japanese English learners (*n* = 37) of the masked form priming lexical decision task in Experiment 1

	English native speakers								Japanese English learners							
	Target words				Target nonwords				Target words				Target nonwords			
	Mean RT		Error rates		Mean RT		Error rates		Mean RT		Error rates		Mean RT		Error rates	
Related	825	(7.60)	4.84	(0.85)	1023	(8.46)	4.17	(0.68)	785	(11.51)	8.11	(1.66)	933	(14.47)	18.47	(2.07)
Unrelated	779	(6.87)	7.80	(0.88)	1005	(8.29)	5.11	(0.87)	837	(11.35)	6.76	(0.96)	930	(13.91)	17.79	(1.59)
Priming	−46		2.96		−18		0.94		52		−1.35		−3		−0.68	

Standard errors are shown in parentheses.

The present study fitted linear-mixed effect (LME) models to the data from native English speakers and Japanese English learners by using the *lme4* package (Bates et al., 2015) in R (Version 2.0), and *p* values were estimated by *lmerTest* package (Kuznetsova et al., 2014). The fixed factors of the model were prime type (related, unrelated), language (English, Japanese), and their interaction. Prime type (related = 0.5, unrelated = −0.5) and L1 (English = 0.5, Japanese = −0.5) were contrast coded. The model had by-subject intercept and slope of prime type, and by-item intercept and slope of the interaction between prime type and L1. We also checked variance inflation factor (VIF) values for each factor (all values were under 2.0). The results indicated that the interaction between prime type and L1 was significant (*t* = 2.69, *p* = .01). Neither the native language predictor (*t* = −0.95, *p* = .35) nor prime type (*t* = −0.05, *p* = .96) were significant. The overall results appear in Appendix Table 6.

**Fig. 1:**
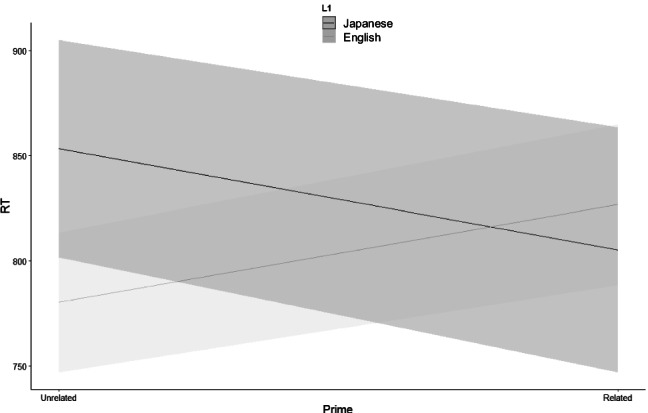
The interaction effect between prime type and L1. The shaded regions are 95% confidence intervals for each condition

The Simple comparisons were conducted between the related and unrelated prime conditions in each L1 group by *emmeans* package (Lenth, 2018), and they demonstrated that the difference was significant based on adjusted *p* value by Tukey method for Japanese learners of English (*z* = −2.13, *p* = .03) but not significant for native English speakers (*z* = 1.71, *p* = .09). These results indicate that null (numerically inhibitory) priming effect was obtained by native English speakers (−46 ms) while significant facilitatory priming effect was obtained by Japanese learners of English (52 ms), as we predicted (see Fig. 1).

Error rates for word targets were also analyzed by using the *lme4* and *lmerTest* packages. The model structure was the same as that for RT data. But the model failed to converge. We therefore changed the optimizer and used the bobyqa function to avoid convergence failure. We also checked VIF values for each factor (all values were under 2.0) The results indicated that the interaction between prime type and L1 (*z* = −1.47, *p* = .14), prime type (*z* = −1.66, *p* = .10), and L1 (*z* = 0.07, *p* = .94) were not significant. The overall results appear in Appendix Table 7.

### Discussion

Experiment 1 was conducted to ensure that (a) adult English native speakers show null or inhibitory priming effects due to the lexical competition and that (b) L2 learners without prior training on the to-be-learned words would show facilitative priming in a masked form priming LDT paradigm. The results clearly confirm these predictions. RTs were slower in the related prime condition than those of the unrelated control condition in the case of native English speakers, which is consistent with findings from previous studies (Davis & Lupker, 2006; Forster & Veres, 1998; Nakayama et al., 2008). One difference between the current findings with native English speakers and earlier studies (Davis & Lupker, 2006; Nakayama et al., 2008) is that the overall response latencies as well as the priming effects are considerably longer in the present study. Although, it is not entirely clear what might account for these differences, neighborhood density values were approximately five times higher in the current study than in the earlier ones. Prior research (Dufour et al., 2007) has found increased response latencies for stimuli with higher neighborhood densities. Despite these differences, RTs were faster in the related prime condition than those of the unrelated control condition in the case of Japanese English learners. This relation was confirmed by the significant two-way interaction between prime type and L1. These results suggest that (a) both prime words and target words are represented in adult native English speakers’ lexicon and caused lexical competition between prime and target words, but (b) since prime words were not represented in the Japanese English learners’ lexicon, presentation of the prime activated only the representation of the target words in the LDT. In other words, the Japanese participants in this experiment did not know the primes, or at least they were not represented in their lexicon, because if these words had been firmly represented in the lexicon, the presentation of the prime should have activated the representation of the prime word itself, which should have caused lexical competition between prime representation and its neighbors. The findings also demonstrate that the experimental stimuli and procedures were valid and that the inhibitory and facilitative priming effects were observed by native English speakers and Japanese English learners, respectively.

Experiment 2 then investigated whether the facilitative priming effect changes as a result of increases in meaning or form processing while learning new L2 words. In addition, Experiment 2 included measures of L1 and L2 recall. These recall tests were conducted both to confirm the findings of Barcroft (2002) with regard to the effects of semantic and structural tasks and as a manipulation check. Regarding the latter of these two, the hypothesis was that if participants were focused on form during the learning phase, they would recall more L2 than because of greater attention to L2 word forms. Conversely, when the learning focused on meaning, they would recall more L1 words due to the memorial benefits of increased semantic processing of the meanings of these L1 words, the forms for which were already known. As was the case in the study by Barcroft (2002), the focus of this experiment was on the comparison of the semantic and form conditions and not that of the no-task (control) condition.

## Experiment 2

### Method

#### Participants

Participants in Experiment 2 were 140 Japanese University learners of English. Among the 140 students, data provided by 32 students were excluded because of their error rates on the semantic or form processing tasks during the entire learning session. Further, data from 19 participants were excluded because of their error rates on the LDT. Although this attrition rate is somewhat higher than in previous studies of PLE, it is important to note that these individuals were learning novel L2 words which is considerably more difficult than the earlier L1 studies. Data from the remaining 89 participants are included in the following analyses (25 participants in the semantic processing condition, 31 participants in the form processing condition, and 33 participants in the control condition). The questionnaire for their background information revealed that most of them started learning English when they were 12 or 13 years old in their school, and the majority of them had at least 6 years of formal instruction of English.^3^ Only one participant reported that he had studied English only for 4 years. Their scores of the TOEIC test indicated that their English proficiency was at the intermediate level. These results suggest that the participants in Experiment 2 belonged to a similar population to those in Experiment 1. The background information of the participants in this experiment appears in Table 4.

**Table 4:** Background information of participants in Experiment 2 (*n* = 89)

	Mean	*SD*	Minimum	Maximum
Age	19.11	0.63	18	22
Age began learning English	12.19	1.88	3	13
Years of formal instruction	6.40	1.01	4	12
TOEIC score	490.86	75.94	320	660
Self-rating:				
Speaking	3.27	1.25	1	6
Listening	3.44	1.48	1	5
Reading	4.01	1.46	1	7
Writing	4.14	1.28	1	7

Self-rating score is on a scale from 0 (*minimum proficiency*) to 10 (*near-native proficiency*). TOEIC (Test of English for International Communication) is a standardized English proficiency test developed by Educational Testing Service

#### Power analysis

The power analysis for Experiment 2 indicated that a sample size of 27 in each condition allowed for the detection of small to moderate (.2 or larger) effects with 80% power. Although the sample size for the semantic condition falls slightly below this value (25 rather than 27), we accepted this small difference for present purposes, especially given the nature of the results (see Results section).

#### Stimuli and design of the experiment

This experiment consisted of a learning session followed by a testing session. In the learning session, there were three word-learning conditions: semantic processing, form processing, and control. In both the free and cued recall tests, we expected that participants focused on form would recall more L2 words, as they would devote a greater percentage of resources to processing L2 word forms. Conversely, we expected that individuals in the semantic learning condition would recall more L1 words as they would focus on meaning, rather than form. Following the recall testing session, there were two counterbalanced conditions in the masked form priming experiment: List 1 and List 2, as was the case with Experiment 1. Therefore, there were six between-subject conditions in total. The stimuli used in Experiment 1 were also used in this experiment. Additional stimuli used in the learning session were selected as follows:

1. Semantically related words to the 24 prime words, which were used in the semantic processing task, were chosen by ensuring that semantic relatedness existed between the two words in the WordNet lexical database (Princeton University, 2010) or Longman *Roget’s Thesaurus* dictionary. When there were several candidates, words whose familiarity ratings were relatively high in Yokokawa’s (2006) English word familiarity rating list for Japanese learners of English were chosen to better guarantee that these semantically related words were known by the participants.
2. Formally related words used in the form processing task were the same as target words in the masked form priming LDT.
3. Distractor words in the semantic and form processing tasks described later in the learning session were words that were not similar to the semantically related words nor formally related words, but had the same word length and similar levels of lexical characteristics of familiarity (Yokokawa, 2006) and frequency (Balota et al., 2007) to the corresponding semantically or formally related words.

The participants were randomly assigned to one of the six between-subject conditions. The counterbalancing in the testing session was achieved in the same way as in Experiment 1.

#### Apparatus and procedure

The same apparatus used in Experiment 1 was used in this experiment. As described above, the experiment consisted of a learning session and a testing session. Before the learning session began, the participants were instructed to remember 24 English words and their L1 translations as best as possible. They were also instructed that they could use any strategies to learn them except for reading them aloud and writing them down. They were also informed that they would be tested afterwards about the L2 words and corresponding L1 translations but were not informed about exactly what kind of tests they would take.

**Fig. 2:**
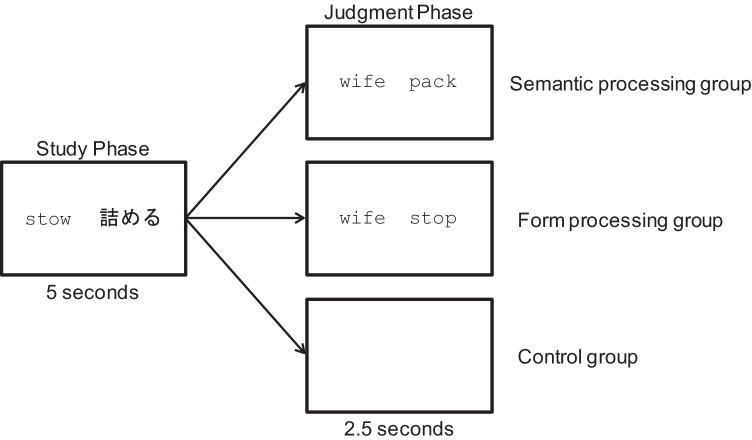
The procedure of the study phase and the judgment phase in the learning session in Experiment 2

Figure 2 displays a schematic of the learning phase. The learning session was composed of four blocks with 24 trials in each block. Each trial consisted of two subparts for each to-be-learned word: the study phase and the judgment phase. In the study phase, a to-be-learned English word and its Japanese translation were shown on a PC monitor for five seconds (e.g., *stow 詰める*). Then, the judgment phase began. For the semantic processing task, two English words were shown on the PC monitor (e.g., *wife pack*), and the participants were asked to indicate which word was more similar to the to-be-learned word’s meaning by pressing the right or left control buttons on the keyboard. For the form processing task, two English words were shown on the monitor (e.g., *wife stop*), and the participants were asked to indicate which word was more similar to the to-be-learned word’s form for 2.5 seconds, again by pressing the right or left control buttons on the keyboard. The distractor words between the two groups were the same (e.g., *wife*). The participants in the control group did not have any additional tasks, and a blank screen was shown for 2.5 seconds. After the 2.5 second judgment phase, the study phase for the next to-be-learned word began and so on. The RTs and error rates of the judgments were measured for each trial.

Participants first experienced a practice session for six words in order to familiarize them with the procedure of the learning session of the experiment. After the practice session, the main experiment began. The participants were exposed to each to-be-learned words four times (24 words × four blocks = 96 trials in total). The order of presentation was pseudo-randomized. That is, the 24 pairs of English to-be-learned words and their Japanese translations were randomly presented to the participants within each block, and the order of four blocks was also randomized for each participant. Further, for the sematic and form processing tasks, the position of the two judged words presented on the monitor was balanced so that words that were semantically or formally similar to the to-be-learned words were shown on the right and left sides equally often.

After the learning session, the testing session began. The testing session consisted of two kinds of tests: four recall tests and the masked form priming LDT. For the first two recall tests, the participants completed L2 and L1 free recall for 2 minutes, respectively, and L1-to-L2 and L2-to-L1 cued recall for 4 minutes respectively. For the L2 free recall, they were instructed to remember and write down as many English to-be-learned words as possible in any order while in the L1 free recall, they were instructed to remember and write down as many Japanese translations of the to-be-learned words as possible in any order. For the L1-to-L2 cued recall, 24 Japanese translations of to-be-learned words were displayed on the monitor, and the participants were asked to write down the corresponding English equivalent, whereas for the L2-to-L1 cued recall, the 24 English to-be-learned words were shown on the monitor, and they were asked to write down the corresponding Japanese translation. Additionally, for the L2-to-L1 cued recall, the participants were asked to indicate if there were any to-be-learned words that they had known before the experiment. Any word(s) that they indicated was (were) not included in the analyses. Note that the free recall tasks were designed to assess the extent to which participants had learned new word form versus new word meaning (free recall in L1 relying largely if not completely on semantically oriented learning and free recall in L2 relying largely if not completely on form-oriented learning), whereas the cued recall tasks were included as measures of vocabulary learning overall given that they rely on the mapping component of L2 vocabulary learning.

After the four recall tests, the masked form priming LDT was administered. For this task, the 24 to-be-learned words in the learning session (e.g., *stow*) were used as primes. Their orthographic neighbors (e.g., *STOP*) were used as targets. As in Experiment 1, the primes were presented for 67 ms.

### Results

Data were not included in the analyses if (1) error rates were more than 10% for the judgment phase in the semantic or form processing tasks (15 participants in the semantic processing group and 17 participants in the form processing group); (2) error rates were more than 20% in the LDT (five participants in the semantic processing group, four participants in the form processing group, and 10 participants in the control group); (3) participants indicated they had prior knowledge about to-be-learned words (37 observations in the total 2,136 responses, 1.73%).

#### Recall tests

**Fig. 3:**
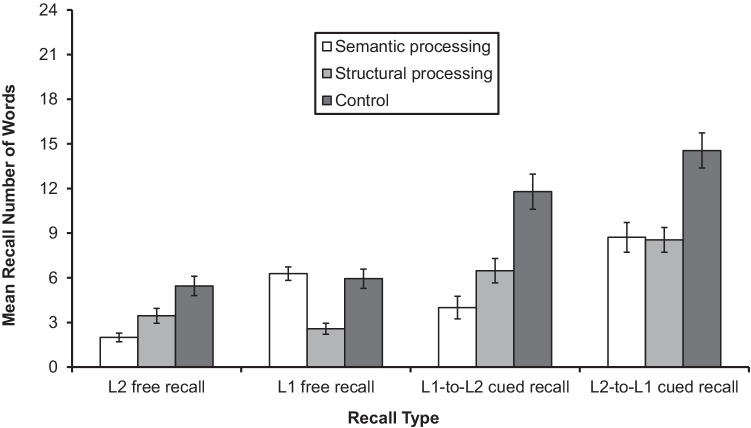
Mean recall number of words by the semantic processing, form processing, and control conditions in the L2 and L1 free recalls and the L1-to-L2 and L2-to-L1 cued recalls. Error bars show standard errors

When scoring the four recall tests, one point was given for each correctly recalled word. All other responses were treated as errors. The results of the L2 free recall, L1 free recall, L1-to-L2 cued recall, and L2-to-L1 cued recall are shown in Fig. 3. The descriptive statistics of the four recall results are shown in Appendix Table 8.

We fitted logit mixed-effects models on the four sets of recall data separately (L2 free recall, L1 free recall, L1-to-L2 cued recall, L2-to-L1 cued recall) using the *lme4* package (Bates et al., 2015) with participants and items as cross-random factors. The models we first fitted to the data had learning condition (semantic, form, control) as the fixed effect and had by-subject intercept and by-item intercept and slope of learning condition. When the model did not converge, we took steps to simplify the random effects structure until the model converged. Based on the prediction of the TOPRA model and results of previous TOPRA-model-based studies (e.g., Barcroft, 2002, 2003, 2004; Kida, 2020; Kida & Barcroft, 2018), our primary focus was different according to the types of processing and types of recall. Therefore, learning condition was dummy coded differently depending on recall types: when the target language was L2 (i.e., L2 free recall and L1-to-L2 cued recall), the form processing condition was the reference level whereas when the target language was L1 (i.e., L1 free recall and L2-to-L1 cued recall), the semantic processing condition was set as reference. We also checked variance inflation factor (VIF) values for each factor (all values were under 2.0).

##### Results for free recall

For L2 free recall, the model included learning condition as a fixed factor and by-subject and by-item intercepts. The results indicated that the difference between semantic processing and form processing (*z* = −2.14, *p* = .03) and between form processing versus control (*z* = 2.53, *p* = .01) were significant. These results indicated that recall of the form processing group was higher than that of the semantic processing group, and that recall of the control group was the highest. Overall results appear in Appendix Table 9.

For L1 free recall, the model included learning condition as a fixed factor and by-subject intercept and by-item intercept and slope of learning condition. The results indicated that the learning condition was significant between semantic processing versus form processing (*z* = −4.95, *p* < .01) but not significant between semantic processing versus control (*z* = −0.60, *p* = .55). These results indicated that, contrary to the results on L2 free recall, recall in the semantic processing group was higher than that of recall in the form processing group. Overall results appear in Appendix Table 10.

##### Results for cued recall

The same model construction procedure was carried out for the cued recall results. For L1-to-L2 cued recall, the model included learning condition as the fixed factor and by-subject intercept and by-item intercept and slope of learning condition. The results indicated that the difference was significant between semantic processing and form processing (*z* = −2.49, *p* = .01) and between form processing and control (*z* = 3.38, *p* < .01). These results indicated that, unlike the results of L2 free recall, recall in the form processing group was higher than that of the semantic processing group, but that recall in the control group was the highest. Overall results can be viewed in Appendix Table 11.

Finally, for L2-to-L1 cued recall, the model included learning condition as the fixed factors, and by-subject intercept, and by-item intercept and slope of learning condition. The results indicated that learning condition was not significant between semantic processing and form processing (*z* = −0.13, *p* > .90) but significant between semantic processing and control (*z* = 3.50, *p* < .01). These results indicated that recall of the control group was higher than that of the semantic and form processing groups. Overall results are depicted in Appendix Table 12.

In sum, the results of the two free recall tests indicated that (a) the form condition outperformed the semantic condition for L2 free recall whereas (b) the semantic condition was better than the form condition for L1 free recall. The results of the two cued recall tests indicated that (c) the form condition outperformed the semantic condition in L1-to-L2 cued recall, whereas (d) no significant difference was found between the semantic and form conditions in L2-to-L1 cued recall. These results generally confirmed that participants in the semantic processing and the form processing tasks engaged in the appropriate type of processing during the learning phase.

#### Masked form priming lexical decision task

##### Results of reaction time

The descriptive statistics of raw RT data for the results of the masked form priming LDT appear in Table 5.

**Table 5:** Mean Reaction times, error rates and their standard errors (appeared in the parentheses) for target words and nonwords in the masked form priming lexical decision task in Experiment 2

	Target words						Target nonwords					
	Reaction time			Error rates			Reaction time			Error rates		
	Related	Unrelated	Diff	Related	Unrelated	Diff	Related	Unrelated	Diff	Related	Unrelated	Diff
Semantic	857 (34)	878 (32)	21	4.17 (0.72)	4.17 (0.93)	0.00	1130 (71)	1143 (75)	13	8.83 (1.16)	7.83 (1.06)	−1.00
Form	781 (27)	816 (25)	35	2.82 (0.78)	4.44 (0.82)	1.62	1004 (54)	1027 (58)	23	6.85 (1.12)	8.06 (1.04)	1.21
Control	885 (42)	864 (36)	−21	3.03 (0.52)	4.92 (0.74)	1.89	1107 (60)	1103 (55)	−4	8.08 (1.02)	8.59 (1.02)	0.51

Analyses were conducted only for the correct responses for word trials. Because the data in Experiment 2 skewed more strongly to the right compared with those in Experiment 1, we applied inverse transformation to the RTs (−1,000/RT) to meet the distributional assumption of LME (Kezilas et al., 2017; Nakayama et al., 2016) before the data analyses reported hereafter, and because we transformed the RT data, we did not exclude outliers in Experiment 2. Fixed factors were learning condition (semantic, form, control), prime type (related, unrelated) and their interaction. Regarding learning condition, the control condition was set at the reference level. Prime type was contrast coded (related = 0.5, unrelated = −0.5). Random effects structures were by-subject intercept and slope of prime, and by-item intercept and slope of the interaction between learning condition and prime type. We also checked VIF values for each factor (all values were under 4.0).

The results indicated that the interaction between learning condition and prime type was not significant (*p* > .10). Learning condition was not significant for the difference between semantic processing and control (*t* = 1.02, *p* = .31) and for between form processing and control (*t* = 0.35, *p* = .73). Additionally, contrary to the results of Experiment 1, prime type was not significant (*t* = 1.12, *p* = .27). Overall results appear in Appendix Table 13.

As in Experiment 1, we conducted simple comparisons between the related and unrelated prime conditions in each learning group. The results indicated that prime type was not significant in any of the learning groups (*z* = 0.62, *p* = .53 for the semantic processing group, *z* = 1.12, *p* = .26 for the form processing group, and *z* = −1.12, *p* = .26 for the control group). These results indicate that, in contrast to the results of Experiment 1, as a result of novel word training during the learning phase, the significant facilitative priming effect disappeared in Experiment 2.

Error rates for word targets were also analyzed by using the *lme4* and *lmerTest* packages. The model structure was the same as that for RT data. But the model failed to converge. As in Experiment 1, we therefore changed the optimizer and used the bobyqa function to avoid convergence failure. We also checked VIF values for each factor (all values were under 3.0) The results indicated that the interaction between learning condition and prime type was not significant (*p* > .20). Learning condition was not significant for the difference between semantic processing and control (*z* = 1.30, *p* = .20) and for between form processing and control (*z* = 0.17, *p* = .87). Prime type was also not significant (*z* = −1.64, *p* = .10). The overall results appear in Appendix Table 14.

### Discussion

These results can be discussed from two perspectives: (a) acquisition of word knowledge for to-be-learned words (lexical configuration) and (b) lexicalization of these words (lexical engagement). In terms of lexical configuration (L2 and L1 free recall and, respectively, L1-to-L2 and L2-to-L1 cued recall), the results revealed that the semantic processing group outperformed the form processing group on the L1 recall test while the opposite results were obtained for the L2 recall test. This double dissociation in the effects of increased semantic versus increased form processing, a pattern also demonstrated by Barcroft (2002), is fully consistent with predictions of the TOPRA model. In addition, the results of the recall tests in the present study revealed that performance of the control group was the highest except in the case of the L1 free recall. One possible reason for this result is that the participants in the control group did not have any specific processing task during the learning phase. Therefore, when they were presented a blank screen in the judgment phase of the learning session (see Fig. 2), it allowed them to rehearse the to-be-learned words and their translations more overall than in the other two conditions. Participants in the semantic processing group and the form processing group, on the other hand, performed an additional task and had less opportunity to rehearse the to-be-learned words and their translations.

As for lexical engagement (masked form priming LDT), the mixed effect models indicated that there was no significant two-way interaction between learning condition and prime type. Simple comparisons indicated no significant priming in all conditions. It is interesting to note that, although not significant, only the control condition demonstrated inhibitory priming numerically. Consistent with these findings, in the original Barcroft (2002) study that included pleasantness ratings (semantic) versus letter counting (formal) versus control (no task), the control condition led to the highest level of vocabulary learning, a finding that, as Barcroft noted, should be interpreted with caution given that no task at all was performed in the control condition, allowing learners simply to attend to the novel words as input more than in the other conditions. During the additional “no-task” time in the control condition, participants could rehearse the L2 words more whereas for the other two conditions they were asked to do specific tasks that apparently got in their way of lexical input processing. These points being made, the present findings are consistent with this pattern observed in the 2002 study but add to it by suggesting that higher levels of vocabulary learning seem to co-occur with increased levels of lexicalization. It is for this reason that patterns consistent with more lexicalization were highest, at least numerically, in the control condition in the present study. Contrary to the findings of some previous studies (e.g., Nakayama & Lupker, 2018; Qiao & Forster, 2017), this experiment demonstrated (a) the PLE in L2 learners functioning in L2 (at least partially) and, moreover, (b) the possibility that lexical competition can operate in L2 learners whose L1 script is different than that of their L2. Possible reasons for these findings are discussed further in the next section.

## General discussion

In this study, Experiment 1 confirmed that null, but numerically inhibitory, priming effects were obtained by native English speakers making lexical decisions on familiar L1 (English) words whereas faciliatory priming effects were obtained by Japanese learners of English who had no previous training on the to-be-acquired English prime words. Experiment 2, in turn, demonstrated that facilitatory priming effects were reduced as a result of the training on the same prime words regardless the types of learning, although different types of learning made difference when it came to learning new L2 words measured at the lexical configuration level (recall) such that form processing was better than semantic processing for L2 word *form* learning (based on L2 free recall) whereas semantic processing was better than form processing for encoding and retention of *semantic* information (based on L1 free recall).

The results of the masked form priming LDT in this study suggest several important points about L2 vocabulary learning and visual word recognition research. First, the results indicated a PLE for Japanese learners of L2 English across the two experiments. When unknown L2 words were used as primes in a masked form priming LDT (Experiment 1), a facilitative priming effect was observed. This effect disappeared when participants learned these words and became familiar with primes (Experiment 2). Therefore, the study provided new evidence regarding how the PLE can be observed in L2 visual word recognition.

The results of Experiment 2 were inconsistent with previous studies that failed to find the PLE after critical L2 word training, such as that of Qiao and Forster (2017). The present findings are partially consistent with those of Nakayama and Lupker (2018) to the extent that we observed facilitation (numerically, although not statistically) between primes and targets in the semantic and form conditions. Nakayama and Lupker (2018) posited that lexical processing in L1 and L2 are different such that facilitatory priming should be observed even when primes are previously known L2 words, and the facilitatory effects observed in Experiment 2 of the present study (at least numerically for the form and semantic processing conditions), are consistent with this interpretation.

Although apparent disparities in the results of these two previous studies (Nakayama & Lupker, 2018; Qiao & Forster, 2017) and the present study may be challenging to explain, several possibilities can be considered. First, regarding the difference between the present study and that of Nakayama and Lupker (2018), it was surprising that the more proficient bilinguals (average TOEIC scores of 849) did not show inhibitory priming effects whereas the less proficient bilinguals in the current study (average TOEIC scores of 491) showed at least numerically inhibitory priming effects (the control group in Experiment 2). It remains an open question how proficiency might affect lexicalization and one that the current study was not designed to address. Nevertheless, one possible reason is that the participants in this study learned new L2 words, which were used as primes in the LDT, immediately before they performed the LDT whereas those in Nakayama and Lupker’s study did not have any prior training because they were highly proficient bilinguals. Therefore, even though the overall L2 proficiency of the participants in Nakayama and Lupker’s study was higher than that of the participants the current study, the primes’ recent activation level should be different which may come into play in the reduction of facilitatory priming effect. Second, regarding the difference between the present study and the study by Qiao and Forster (2017), the training procedure may contribute to the difference. In Qiao and Forster (2017), the participants first saw each to-be-learned word on the PC screen for 1,660 ms, then its corresponding picture appeared on it for 1,660 ms, followed by its definition for 3,320 ms. They then had word–picture matching, picture–word matching, and word–definition matching practice in which a word or picture was shown on the top of the screen and two alternatives were presented and participants were asked to select the appropriate one. These matching practices may not have engaged form-based processing to the same extent as in the current study because participants could perform these tasks just by indicating which picture or definition they saw during the learning phase. In this way, they did not have to remember the to-be-learned word forms. In the present study, on the other hand, the participants were asked to remember L2 word forms and their L1 translations. This difference might play role in the reduction of the facilitatory priming effect.

Now let us also consider further implications of the combined findings of the study. These include (a) a facilitatory priming effect among Japanese learners of English without critical word training (Experiment 1), (b) null effects with critical word training (Experiment 2), and (c) inhibitory priming among native speakers of English (Experiment 1) while using the same experimental stimuli and procedure. This pattern of results points to a developmental trajectory of the L2 lexicon in which facilitatory priming is obtained before L2 words become lexicalized, first partially and then more fully, over time. Studying the prime words in Experiment 2 may have initiated lexical configuration for the Japanese learners of English, but integration into the mental lexicon was not as strong or complete as for L1 English speakers in Experiment 1. One interpretation of these results is that the learning session helped L2 learners to become more familiar with the to-be-learned words and their meanings to the degree that a PLE was found, but the learning was not complete or thorough enough to yield inhibitory effects in the LDT. The failure to find significant inhibitory priming in Experiment 2 may be specific to the training paradigm this study used, however. The participants were exposed to each word only four times (24 words × four blocks = 96 trials in total) in about 15 minutes, which appears to be insufficient for more complete lexicalization. This interpretation is consistent with previous studies demonstrating that (a) lexicalization of new L2 words requires multiple study/learning sessions (e.g., Elgort, 2011; Qiao & Forster, 2013) or sleep consolidation (Dumay & Gaskell, 2007; Wang et al., 2017).

One way to conceptualize the process of lexicalization based on the current findings is as a continuum of integration within the lexicon. Initially, unfamiliar L2 words exhibit neither lexical configuration nor lexical engagement. Instead, these novel L2 words are represented as form-based phonetic sequences^4^ in working memory, and priming is facilitative because they activate similar form-based sequences within the lexicon. Small amounts of training, as in Experiment 2, can familiarize individuals with the novel L2 words and initiate lexical configuration. During this phase, representations of the form-based sequences may transition from working to long-term memory but are minimally integrated within the mental lexicon. Masked form priming at this stage will likely show either reduced facilitation (relative to the initial stage) or neither facilitation nor inhibition. After additional exposure or training, the phonetic sequences undergo lexical engagement and are increasingly integrated within the mental lexicon. Masked form priming at this point will be inhibitory as the now integrated representations compete with existing lexical items.

An interesting implication of the current study is that both semantically oriented and form oriented tasks may slow this process of lexical engagement, relative to a condition (the control condition), at least numerically, in which participants did not have to perform any task. Although the absence of significant differences between the form and semantic learning tasks must  be viewed cautiously given that participants received only four learning trials for each word, it was only the control condition that showed evidence of inhibitory priming. That is, rather than form-based tasks facilitating lexicalization, as originally predicted, it may be that simply allowing learners to rehearse or practice the association between L1 and L2 will more strongly increase the probability of moving toward the lexical engagement end of the continuum.

### Limitations and future research

Two potential limitations of the current study are that (a) the number of stimuli in each condition (12) was relatively small compared with previous studies that have used the prime lexicality effect to examine lexicalization of novel words (Qiao & Forster, 2013, 2017) and that (b) word targets in the masked form priming were also used as stimuli in the judgment task for the form (but not semantic or control) learning conditions. Regarding the first of these, our decision to use 12 items per condition was based on the study of lexical engagement and lexical configuration by Leach and Samuel (2007). Regarding the second, repetition of the word targets from the form learning phase as targets in the masked form priming phase may indeed have affected the amount and significance of priming. It would be informative if future studies of the PLE as a measure of L2 lexicalization examined differences between form and semantic focused learning with increased number of stimuli, at least to levels similar to those used in other studies (Qiao & Forster, 2017) and without repetition between the learning and LDT phases. Another limitation concerns the qualitatively different nature of the no-task control condition in the present study (and in the study by Barcroft, 2002) in that this condition allowed participants simply to continue attempting to learn new L2 words in an undeterred manner. Future studies could ask participants to complete a different type of task, such as a nonlinguistic task (e.g., completing math problems), so as to direct learners’ processing in a different way, one that restricts input processing in a specific way, as do the semantic and form tasks, while allowing for a new type of comparison between all the three conditions. A third task of this nature, which would be “no task” in the sense of not being a language-related task (neither form-oriented or meaning-oriented), could allow for new comparisons that would be informative both in terms of theory and practice, such as with regard to the overall amount of vocabulary learning that takes place when a learner is distracted completely by a nonlinguistic task versus performing a semantic or form-oriented task.

In addition to addressing these three issues, future studies with more extensive training regimens also can be conducted to continue to explore how quickly L2 learners can begin to lexicalize L2 words when they are engaged in different types of training/instructional programs for L2 vocabulary learning. These programs can include and expand upon the types of training assessed in this study, such as semantically versus formally elaborative training, but they can also go further to explore other learning contexts for L2 vocabulary learning, such as classroom-based versus study-abroad contexts, and systematically explore the effects of other variables on the degree to which L2 learners are able to lexicalize words and different types of lexical phrases, including variables that relate to lexical input processing, such as frequency of exposure to lexical items, number of retrieval opportunities, and the extent to which the manner in which a lexical item is presented offers opportunities for lexical inferencing.

### Summary and conclusion

In sum, this study investigated the lexicalization of new L2 words in the masked form LDT. In Experiment 1, null priming effects were observed for native English speakers and faciliatory priming among adult Japanese English learners without critical word training. Experiment 2 investigated effects of increases in semantic and form processing of new L2 words on memory for these words based on recall tests and on lexicalization of newly learned words in the mental lexicon by using a masked form priming LDT. The results of the recall tests demonstrated that semantic processing increased memory for new word meanings while decreasing memory for new word forms. Form processing, on the other hand increased learning of new word forms while decreasing semantically oriented memory, which in this case corresponded to memory for having been exposed to the previously acquired (known L1) words. These results are consistent with the predictions of the TOPRA model, pointing to the dissociability of word forms and word meanings during L2 vocabulary learning. Finally, the PLE effect revealed across the two experiments suggests that newly learned L2 words can become increasingly integrated over time, passing thresholds that have come to be associated with lexicalization, as part of L2 learners’ constantly evolving lexicosemantic systems. This is especially true if, as in the control condition of the current study, participants are allowed to process the novel words without the need for additional form or semantic processing.

## Data Availability

The datasets generated during and/or analyzed in the current research are available in the Open Science Framework repository (https://osf.io/f6e2m/).

## Appendix 1

**Table 6:** The results of the mixed-effect models for RT data in Experiment 1

	Fixed effects				Random effects	
					By Subject	By Item
	Estimate	*SE*	*t*	*p*	*SD*	*SD*
Intercept	816.39	14.77	55.27	<.01	109.22	43.12
Prime	−0.90	17.94	−0.05	.96	155.00	18.47
L1	−25.81	27.26	−0.95	.35	–	65.82
Prime × L1	94.51	35.19	2.69	.01	–	14.22

*Note*. L1, *SE*, and *SD* refer to native language, standard error, and standard deviation, respectively.

**Table 7:** The results of the mixed-effect models for error data in Experiment 1

	Fixed effects				Random effects	
					By subject	By item
	Estimate	*SE*	*z*	*p*	*SD*	*SD*
Intercept	−2.90	0.16	−17.60	<.01	0.46	0.45
Prime	−0.45	0.27	−1.66	.10	0.60	0.42
L1	0.02	0.29	0.07	.94	–	0.76
Prime × L1	−0.66	0.45	−1.47	.14	–	0.34

*Note*. L1, *SE*, and *SD* refer to native language, standard error, and standard deviation, respectively.

## Appendix 2

**Table 8:** Percentile scores and their 95% confidence intervals (CIs) in all test types under all conditions in Experiment 2

	L2 free recall		L1 free recall		L1-to-L2 cued recall		L2-to-L1 cued recall	
	%	95% CI	%	95% CI	%	95% CI	%	95% CI
Semantic	8.33	[6.24, 10.84]	26.17	[22.69, 29.88]	16.67	[13.77, 19.89]	36.33	[32.48, 40.32]
Form	14.38	[11.94, 17.11]	10.75	[8.62, 13.20]	27.02	[23.85, 30.36]	35.62	[32.17, 39.18]
Control	22.73	[19.85, 25.81]	24.75	[21.78, 27.91]	49.12	[45.58, 52.66]	60.61	[57.11, 64.03]

**Table 9:** The results of the mixed-effect model for L2 free recall in Experiment 2

	Fixed effects				Random effects	
					By subject	By item
	Estimate	*SE*	*z*	*p*	*SD*	*SD*
Intercept	−2.03	0.20	−10.32	<.01	0.77	0.35
Semantic vs. Form	−0.60	0.28	−2.14	.03	–	–
Form vs. Control	0.61	0.24	2.52	.01	–	–

*Note*. *SE* and *SD* refer to standard error and standard deviation, respectively.

**Table 10:** The results of the mixed-effect model for L1 free recall in Experiment 2

	Fixed effects				Random effects	
					By subject	By item
	Estimate	*SE*	*z*	*p*	*SD*	*SD*
Intercept	−1.12	0.17	−6.55	<.01	0.57	0.41
Semantic vs. Form	−1.26	0.25	−4.95	<.01	–	0.54
Semantic vs. Control	−0.12	0.20	−0.60	.55	–	–

*Note*. *SE* and *SD* refer to standard error and standard deviation, respectively.

**Table 11:** The results of the mixed-effect model for L1-to-L2 cued recall in Experiment 2

	Fixed effects				Random effects	
					By subject	By item
	Estimate	*SE*	*z*	*p*	*SD*	*SD*
Intercept	−1.34	0.30	−4.49	<.01	1.33	0.71
Semantic vs. Form	−1.04	0.42	−2.49	.01	–	0.47
Form vs. Control	1.22	0.36	−3.38	<.01	–	–

*Note*. *SE* and *SD* refer to standard error and standard deviation, respectively.

**Table 12:** The results of the mixed-effect model for L2-to-L1 cued recall in Experiment 2

	Fixed effects				Random effects	
					By subject	By item
	Estimate	*SE*	*z*	*p*	*SD*	*SD*
Intercept	−0.83	0.37	−2.56	.02	1.35	1.12
Semantic vs. Form	−0.05	0.39	−0.13	.90	–	–
Semantic vs. Control	1.43	0.41	3.50	<.01	–	0.64

*Note*. *SE* and *SD* refer to standard error and standard deviation, respectively.

## Appendix 3

**Table 13:** The results of the mixed-effect model for RT data in Experiment 2

	Fixed effects				Random effects	
					By subject	By item
	Estimate	*SE*	*t*	*p*	*SD*	*SD*
Intercept	−1.39	0.09	−15.90	<.01	0.42	0.21
Semantic vs. Control	0.12	0.12	1.02	.31	–	0.09
Form vs. Control	0.04	0.11	0.35	.73	–	0.11
Prime	0.09	0.08	1.12	.27	0.21	0.24
Semantic × Prime	−0.13	0.10	−1.27	.21	–	0.23
Form × Prime	−0.15	0.10	−1.55	.13	–	0.25

*Note*. SE and SD refer to standard error and standard deviation, respectively.

**Table 14:** The results of the mixed-effect model for error data in Experiment 2

	Fixed effects				Random effects	
					By subject	By item
	Estimate	*SE*	*z*	*p*	*SD*	*SD*
Intercept	−3.51	0.40	−8.72	< .01	0.41	1.20
Semantic vs. Control	0.53	0.41	1.30	.20	–	0.66
Form vs. Control	0.08	0.48	0.17	.87	–	1.12
Prime	−1.08	0.66	−1.64	.10	0.59	1.09
Semantic × Prime	0.91	0.76	1.19	.23	–	0.70
Form × Prime	0.24	0.83	0.29	.77	–	0.83

*Note*. *SE* and *SD* refer to standard error and standard deviation, respectively.

## Appendix 4

**Table:**

Target words	Related primes (to-be-learned words in Experiment 2)	Control primes	Japanese translations	Meaning related words	Distractor words
ABOVE	abode	flirt	住居	home	fight
ALLOW	aglow	furry	輝く	bright	funny
BANK	bunk	yarn	寝台	bed	gold
COLD	colt	kick	子馬	horse	rule
KNOCK	knack	flute	才能	talent	marry
LACK	lank	tend	細い	thin	busy
LUCKY	mucky	sheet	汚い	dirty	sound
OPEN	omen	flux	前兆	sign	land
PLANT	slant	curly	観点	angle	drive
SKIN	skid	puff	滑る	slip	fact
STOP	stow	lava	詰める	pack	wife
WATCH	latch	bells	かんぬき	lock	treat
AWAY	awry	edit	誤った	wrong	under
BASE	bash	twin	お祝い	party	cost
BLOW	blob	jive	染み	spot	nice
CHASE	chasm	wired	穴	hole	bird
CODE	coda	sink	終章	end	milk
COUNT	fount	media	源泉	source	heavy
HIGH	nigh	romp	近い	near	race
HOUSE	douse	inch	濡れる	wet	hard
MAGIC	manic	dwell	熱狂的な	active	floor
REACH	leach	towel	ろ過する	filter	mouth
SALE	sane	pout	正気の	reasonable	light
WATER	wager	spice	賭ける	bet	quick

## Footnotes

1 Given that the main purpose of the current study was to compare response latencies for the unrelated and related conditions using the newly “learned” words as primes, we elected not to include word primes with nonword targets (and to avoid additional time burden on our participants). As the identical comparisons were made across all three learning conditions, we do not believe that using only nonword primes with nonword targets would have altered the pattern of results.

2 The duration of prime (67 ms) in this study was based on a previous L2 lexical competition study (Nakayama & Lupker, 2018) who argued that 67 ms were long enough for L2 different-script L2 learners to produce activation of the primes, but short enough not to be visible.

3 Self-rating information from 19 participants was not available due to a procedural error.

4 The present study was not designed to discern how the Japanese English bilinguals processed each visual presentation phonetically because it was conducted in the written mode; however, a future spoken-word version partial replication could be very profitable in improving our understanding in this area.
